# Isolation and Characterization of a Phosphorus-Solubilizing Bacterium from Rhizosphere Soils and Its Colonization of Chinese Cabbage (*Brassica campestris* ssp. *chinensis*)

**DOI:** 10.3389/fmicb.2017.01270

**Published:** 2017-07-26

**Authors:** Zhen Wang, Guoyi Xu, Pengda Ma, Yanbing Lin, Xiangna Yang, Cuiling Cao

**Affiliations:** College of Life Sciences, Northwest A&F University Yangling, China

**Keywords:** phosphate-solubilizing bacteria, phosphorus, Chinese cabbage, colonization, rhizosphere

## Abstract

Phosphate-solubilizing bacteria (PSB) can promote the dissolution of insoluble phosphorus (P) in soil, enhancing the availability of soluble P. Thus, their application can reduce the consumption of fertilizer and aid in sustainable agricultural development. From the rhizosphere of Chinese cabbage plants grown in Yangling, we isolated a strain of PSB (YL6) with a strong ability to dissolve P and showed that this strain promoted the growth of these plants under field conditions. However, systematic research on the colonization of bacteria in the plant rhizosphere remains deficient. Thus, to further study the effects of PSB on plant growth, in this study, green fluorescent protein (GFP) was used to study the colonization of YL6 on Chinese cabbage roots. GFP expression had little effect on the ability of YL6 to grow and solubilize P. In addition, the GFP-expressing strain stably colonized the Chinese cabbage rhizosphere (the number of colonizing bacteria in the rhizosphere soil was 4.9 lg CFU/g). Using fluorescence microscopy, we observed a high abundance of YL6-GFP bacteria at the Chinese cabbage root cap and meristematic zone, as well as in the root hairs and hypocotyl epidermal cells. High quantities of GFP-expressing bacteria were recovered from Chinese cabbage plants during different planting periods for further observation, indicating that YL6-GFP had the ability to endogenously colonize the plants. This study has laid a solid and significant foundation for further research on how PSB affects the physiological processes in Chinese cabbage to promote plant growth.

## Introduction

After nitrogen, phosphorus (P) is the second most essential macronutrient for plant growth (Takahashi and Anwar, 2007). P regulates the functions and activities of many enzymes and affects plant physiology (Schachtman et al., 1998; Bucher, 2007). P deficiency is commonly considered to be an important limiting factor in agricultural production, particularly in China (Yin, 2011). Therefore, massive amounts of phosphate fertilizers have been applied to optimize plant yields in the past century. However, a large portion of the phosphate fertilizer is unavailable to plants because large amounts of P rapidly become immobilized and insoluble (Shenoy and Kalagudi, 2005; Viruel et al., 2011). P can be easily dissolved in water, which leads to the loss of P in the soil and the eutrophication of nearby water bodies, resulting in other consequential pollution problems. In addition, the excessive application of phosphate fertilizers may cause heavy metal accumulation in soil because most of the phosphate fertilizers contain a variety of heavy metals (Ulén et al., 2007). Thus, environmentally friendly substitutes for phosphate fertilizers must urgently be found to avoid the adverse effects of agricultural production (Singh and Reddy, 2011).

In rhizosphere soil, a large number of plant-growth-promoting rhizobacteria (PGPR) are present, particularly phosphate-solubilizing bacteria (PSB), which have the ability to promote plant growth and increase plant production (Fürnkranz et al., 2009; Hanif et al., 2015). These bacteria have the ability to convert insoluble P into an available form (Hanif et al., 2015). The use of PSB in sustainable agriculture has increasingly attracted the attention of scientists (Hameeda et al., 2008). At present, identified PSB primarily belong to the *Pseudomonas, Bacillus, Mycobacterium*, and *Enterobacter* genera, among others (Hanif et al., 2015; Li et al., 2015). Two types of P-solubilization mechanisms in PSB are generally believed to occur. One is the release of organic acids by PSB to dissolve insoluble P, including malic, lactic, acetic, citric, and succinic acids (RodríGuez and Fraga, 1999). The second mechanism is the secretion of enzymes by PSB that are able to degrade insoluble P. Some PSB can even produce plant growth regulators, or phytohormones, such as indole acetic acid (IAA) and gibberellin (GA) (Seo and Park, 2009; Babu et al., 2015).

Many studies have shown that PSB can promote plant growth. PSB can improve the growth of lentil plants with respect to plant height, root length, number of branches, number of seeds per pod, and number of seeds per plant by secreting plant growth promoting substances (Singh et al., 2011). Zhao et al. (2014) isolated 12 strains of PSB in Sichuan and found a strain (SCAK0330), later identified as *Burkholderia cepacia*, that could solubilize over 450 μg/mL of P, promote the growth of maize, and reduce the infection of small plaque-causing bacteria in corn, thus showing its utility as a biofertilizer and a biocontrol agent. Yu et al. (2011) found that the ability of PSB to solubilize P is negatively affected by the pH of the soil and that the absorption of N and P by walnut seedlings and the P-solubilization activity of soil significantly improved after the application PSB. In addition, the number of PSB that colonized the rhizosphere was found to be significantly increased, and the soil pH was reduced. Many more of these microorganisms are present in rhizosphere than in non-rhizosphere soil (Kucey et al., 1989). Thus, the ability of PSB to colonize the rhizosphere determines its overall P-solubilization capacity and its ability to promote plant growth (Ramachandran et al., 2007). Until now, the majority of studies have only focused on the isolation and identification of such bacteria and on other aspects; however, there is still a lack of information on PSB colonization of the rhizosphere, survival patterns, and on the mechanism of P-dissolution, all of which require further systematic studies.

Green fluorescent protein (GFP), which was first isolated from jellyfish (*Aequorea*), is widely used as a visual reporter in cell and molecular biology (Chalfie, 1994; Gamalero et al., 2004). This protein is used in research for the colonization of microorganisms because they allow for cells to be easily observed by fluorescence microscopy (Prosser, 1994; Zimmer, 2002). At present, GFP is commonly used with bacteria, such as *Bacillus* and *Pseudomonas* species, in studies of microorganism colonization (Fernández et al., 2012; Ji et al., 2014; Padda et al., 2015). Antibiotic resistance has been used as a marker to quantify the colonization dynamics of microbes in plant-root systems. Characteristics of the antibiotic markers selected for the PGPR must be determined prior to being used because some soil microorganisms produce different types of antibiotics. A fluorescent and apramycin-resistant *Streptomyces* strain was observed in roots, and its organization was confirmed as an endogenous bacterium (Bonaldi et al., 2015). However, few studies on the colonization of GFP-labeled PSB in Chinese cabbage have been reported.

In this study, we isolated a strain of PSB (Yangling 6, YL6) from the rhizosphere soil of Chinese cabbage (*Brassica campestris* ssp. *chinensis* var. *communis Tsen et Lee*), which proved capable of solubilizing P. The application of YL6 dramatically reduced the need to use chemical fertilizers and aided in sustainable agricultural development in the study area. Chinese cabbage, a traditional Chinese vegetable, is rich in calcium, potassium, and vitamin C, providing basic nutrients for human dietary needs (Pokluda, 2008). The short growth cycle of Chinese cabbage makes it a perfect experimental model system. Thus, in this study, the effects of YL6 on the growth of Chinese cabbage under field conditions were investigated. To further explore the localization and colonization of YL6 in Chinese cabbage roots and rhizosphere, a GFP-labeled YL6 strain was used.

## Materials and Methods

### Isolation and Identification of Bacteria

The study site (N 34°17′9.81″, E 108°04′9.61″) was located in an experimental field of Northwest A&F University, Yangling, Shaanxi, China. The soil samples were collected from the rhizosphere soil of Chinese cabbage at a depth of 5–15 cm. Rhizospheric bacteria were isolated from one gram of rhizosphere soil by serial dilution plating (Somasegaran and Hoben, 1994) on Pikovskaya’s agar plates containing tricalcium phosphate (TCP 10 g/L) (Ri, 1948). The plates were incubated at 28 ± 2°C until halos/zones appeared around the colonies. Then, the bacterial strains were purified, and the growth of isolates on screening media was used to compare the P-solubilization abilities. Among several isolated bacterial strains, YL6 showed a remarkable ability to dissolve in organic P and was selected for further studies.

Yangling 6 was biochemically characterized and identified using Bergey’s manual of systematic bacteriology (Sneath et al., 1986). YL6 DNA was obtained by the lysozyme-SDS-phenol/chloroform method (Sambrook et al., 1989; Chong, 2001) and was used as template DNA in PCR to amplify the 16S rRNA gene using the universal bacterial 16S rRNA primers (forward: 5′-CGGGATCCAGAGTTTGATCCTGGTCAGAACGAACGCT-3′ and reverse: 5′-CGGGATCCTACGGCTACCTTGTTACGACTTCACCCC-3′) according to Tan et al. (1997). The 16S rRNA gene PCR product was confirmed on a 1% agarose gel and purified using a DNA Gel Extraction Kit (Qiagen); then, it was ligated to a pMD18-Tvector (Takara, Dalian) and transformed into *Escherichia coli* DH5α cells prior to sequencing (Invitrogen^TM^, Shanghai, China). The derived sequence was compared to known bacterial 16S rRNA gene sequences in the NCBI GenBank database using the BLAST algorithm to identify the isolate.

### Determination of P-Solubilization and Organic Acid Production by YL6

A single colony of each purified isolate was spotted on Pikovskaya’s agar plates, incubated at 28 ± 2°C, and observed for up to 48 h for halo/zone formation (Ramachandran et al., 2007). To quantify the soluble P and organic acids present in culture media, 100 mL of Pikovskaya’s broth containing a single colony of each isolate was incubated in a 250-mL Erlenmeyer flask at 28 ± 2°C for 72 h in a shaker at 180 rpm; this experiment was performed in triplicate. After 24, 48, and 72 h of growth, 20 mL of each culture was harvested and centrifuged at 13,000 × *g* for 10 min to obtain cell-free supernatants. The supernatants were analyzed for soluble P content and phosphatase activity by the molybdate blue (Mo-blue) (Murphy and Riley, 1962) and sodium phosphate methods, with the absorbance measured at 700 and 405 nm using a spectrophotometer (Mclachlan, 1980), respectively. The pH was measured using a pH meter. All measurements were performed in triplicate.

For the high-performance liquid chromatography (HPLC) analysis of organic acids, the cell-free supernatants were filtered through 0.45-μm nylon filters (Millipore, United States). Then, 20 μL was injected into the HPLC instrument (Essentia LC-15C, Japan), equipped with a C-18 column, and run at a flow rate of 1 mL/min using a 90:10 (v/v) methanol:phosphate buffer (10 mmol/L), pH 2.7, as the mobile phase; this process was monitored at 210 nm (Paavilainen and Korpela, 1993; Qin et al., 2010; Fernández et al., 2012).

### Field Experiments

The objective of this work was to evaluate the phosphate mineralization potential of strain YL6 and its effect on Chinese cabbage growth. The field experiment was performed in an experimental field in the North Campus of Northwest A&F University. Four treatments were performed, including YL6 fermentation broth (YL6), Pikovskaya’s broth (CK1), phosphate fertilizer (CK2), and water (CK0). The test field was divided into 12 plots, each 2 m^2^ × 2 m^2^. The experiment was conducted in a randomized complete block design with three replications. Before planting, the soil was turned and sowed manually by the dibbling method, with three seeds per hole, with a between-row and between-plant distance of 30 and 8 cm, respectively, in every plot. The Chinese cabbages were harvested after 45 days. Agronomic characteristics, such as plant height, leaf number, root length and volume, and shoot and root dry weight were measured.

### Physical and Chemical Properties of Rhizosphere Soils

The rhizosphere soils were aseptically separated from the roots to assess the physical and chemical properties from each plant treatment. The pH was measured in a 2:1 water/soil suspension with a pH meter (Jackson, 1958). Soluble P was extracted by the bicarbonate method and was then analyzed by the molybdate blue method (Colwell, 1963). The organic matter content was measured using the potassium dichromate colorimetric method (Nelson and Sommers, 1996). The available potassium content was determined using the flame photometer method (Gammon, 1951). The content of nitrate and ammonium nitrogen extracted by a potassium chloride solution was determined with an Auto Analyzer 3 (AA3) (SEAL, Germany) (Crooke and Simpson, 1971; Best, 1976).

### Colonies of YL6 in Rhizosphere Soils

The roots from three replicates per treatment were harvested with the adhered potting soil. Then, 2 g of rhizosphere soil was added to sterile 50-mL Erlenmeyer flasks with 18 mL of sterile water. The flasks were shaken on a rotary shaker for 20 min at 200 rpm, and then 100 μL of soil suspension was plated in serial dilutions on Pikovskaya’s plates. The colonies showing a clear zone of TCP dissolution were enumerated at the end of the incubation period (Tao et al., 2008).

### GFP Labeling of YL6

#### Bacterial Strains, Plasmids, and Growth Conditions

*Escherichia coli* DH5α was used as the donor strain for the conjugation of plasmid pMP2444, which carried a GFP gene and a gentamicin (Gm) resistance marker. The *E. coli* strain HB101 (harboring the helper plasmid pK2013) was used to transport the pMP2444 plasmid. Both pMP2444 and pK2013 were kindly provided by Prof. Gehong Wei, Northwest A&F University, Yangling, Shaanxi, China. The two strains were grown on Luria-Bertani (LB) medium at 37°C (Gilbertson et al., 2007). A triparental conjugation was used to introduce the pMP2444 plasmid into YL6 as described by Poonguzhali et al. (2008). Gentamicin (50 μg/mL) was added to select the recombinant GFP strains as previously described (Gilbertson et al., 2007). A single colony with a transparent halo was picked and used as a template for PCR identification using the primers M13F (5′-TGTAAAACGACGGCCAGT-3′) and M13R (5′-CAGGAAACAGCTATGAC-3′). After an initial denaturation for 5 min at 95°C, 35 cycles were performed as follows: denaturation at 95°C for 30 s, annealing at 58°C for 30 s, and extension at 72°C for 30 s. A final extension at 72°C for 2 min was performed. The PCR products were detected by agarose gel electrophoresis. Transconjugants isolated from the mating assays were assessed for morphology and motility, and green fluorescence was checked by epifluorescence microscopy (Olympus BX53, Japan) using a filter for green signal (FITC, 488 nm). The obtained GFP-expressing strain was named YL6-GFP.

#### Comparison of the Parental (YL6) and GFP-Expressing (YL6-GFP) Strains

The growth curves of YL6 and YL6-GFP were determined by optical density measurements at 600 nm (OD_600_). Stationary phase cultures were adjusted to an OD_600_ of 1.0 and were then diluted 1:100 in LB and incubated at 25°C with shaking at 200 rpm. Bacterial concentrations (OD_600_) were estimated at 1, 2, 3, 4, 5, 6, 7, 8, 9, 10, 11, and 12 h in duplicate. Soluble P and organic acid contents of YL6-GFP and YL6 cultures utilized the same methods.

#### Colonization Dynamics of YL6-GFP in Natural Soil

Soil samples were inoculated with 3, 6, and 9% (v/w) of the YL6 or YL6-GFP fermentation cultures (8.3 lg CFU/mL) in plastic pots (12 cm × 10 cm × 8 cm) in triplicate and were then cultured in the natural environment (25°C, 22% relative humidity) in April. The number of YL6-GFP bacteria were estimated by serial dilution plating techniques on Gm-LB medium at 0, 7, 14, and 21 days. The plates were incubated at 30°C for 3 days. YL6-GFP colonies were counted, and the number of bacteria was expressed as the lg CFU/g of rhizosphere soil dry weight.

#### Pot Experiment

The fermentation broth of YL6 and YL6-GFP (8.3 lg CFU/mL) was prepared prior to use. The Chinese cabbage seeds were planted in pots (24 cm × 17 cm × 16 cm) with 3 kg of natural soil. Ten Chinese cabbage seeds were planted in each pot, with only five plants/pot kept after seedlings reached the trefoil stage. YL6 (YL6) and YL6-GFP (YL6-GFP) fermentation cultures, Pikovskaya’s medium (CK1) and H_2_O (CK0) were applied to the plants at 9% (v/w). The plants were grown under natural growth conditions, maintaining a 20% absolute water content. Twenty days after the application, the plants were harvested, and the plant growth, agronomic characteristics, and soil soluble P content were measured. The number of YL6-GFP cells in the rhizosphere soil was estimated using the serial dilution plating technique on Gm-LB medium at 0, 5, 10, 20, and 30 days.

#### Fluorescence Microscopy Observations of Chinese Cabbage Root Colonization after YL6-GFP Application

The seeds of Chinese cabbages were sterilized with 2 mL of 0.2% NaClO for 5 min and were then rinsed in 70% ethanol for 30 s. Subsequently, the seeds were washed five times with sterile water (Narendra et al., 2015). The sterilized seeds were soaked in 8.3 lg CFU/mL YL6-GFP suspensions for 30 min. Then, 50 seeds were incubated in water media and kept at 25 ± 2°C under 2000 lx of illumination intensity with a 16 h light period. Aseptic culture techniques were used to avoid contamination by other microorganisms. Fluorescence microscopy was used to visualize the internal colonization of fresh Chinese cabbage roots that had been sliced into 1 cm fragments and mounted on glass slides on the first or third day after planting to confirm the endophytic behavior.

#### Seed Inoculation and the Growth of Chinese Cabbage Plants

The surface-sterilized seeds were immersed in the YL6-GFP fermentation culture for 30 min. The seeds were planted in glass bottles (6 cm × 6 cm × 9 cm) including sphagnum substrate (Pindstrup, Denmark) in a growth chamber (25°C, 55% relative humidity in a 16/8 h day/night cycle). A bottle that was only inoculated with water was used as the inoculated control. One-, two-, and three-week-old plants were used to verify the ability of the YL6-GFP to colonize in Chinese cabbage.

### Statistical Analysis

Regression and correlation analyses were performed to determine the relationship among the changes in pH of medium, soluble P, and organic acid production using the SPSS software package version 16.0 (SPSS, Inc., Chicago, IL, United States). Data were assessed using analysis of variance (ANOVA), and least significant difference (LSD) tests were used at a 5% probability to compare the differences among treatments (Steel et al., 1981).

## Results

### Isolation and Strain Identification

The bacterial strain YL6 (*Bacillus cereus*) isolated from rhizosphere soils was a gram-positive (**Supplementary Figure S1**), motile bacterium with a rod-shaped cell morphology. Colonies of this strain on agar plates were creamy and milky white with regular margins. To identify the isolated strain, a BLAST search of the 16S rRNA gene sequence was performed. The 16S rRNA gene sequence of strain YL6 was submitted to the NCBI GenBank and get accession number KX580383. The results of the BLASTn search showed that the YL6 16S rRNA gene sequence was 100% identical to a *B. cereus* strain (Acc. no. KT719671).

### Evaluation of YL6 P-Solubilization and Organic Acid Production

P-solubilization was tested by inoculating YL6 onto Pikovskaya’s agar plates for 48 h and measuring the diameter of the halo/zone around a bacterial colony. The diameter of the YL6 colony was 0.7 mm, and the diameter of the halo/zone was 0.4 mm (**Supplementary Figure S2**). To further analyze the P-solubilization activity of YL6, the soluble P content in liquid cultures was evaluated by the Mo-blue method. The amount of P solubilized by YL6 varied from 10.1 to 13.6 μg/mL (**Table 1**). At 48 h, the soluble P content reached 13.6 μg/mL, at which time the lowest pH value of the culture (4.0) was observed. At 24, 48, and 72 h, as the soluble P increased in the liquid cultures, the activity of acid phosphatase was changed too. HPLC analysis showed the presence of different organic acids, such as tartaric, acetic, citric, ascorbic, and lactic acids, during the P- solubilization by YL6 (**Table 1**).

**Table 1:** Effects of the biological characteristics of the original strain (Yangling 6, YL6) and the green fluorescent protein (GFP)-expressing strain (YL6-GFP) at different times.

Treatments	Tartaric acid μg/mL	Lactic acid μg/mL	Acetic acid μg/mL	Citric acid μg/mL	Ascorbic acid μg/mL	Alkaline phosphatase μg/h.ml^-1^	Acid phosphatase μg/h.ml^-1^	Soluble P mg/kg	pH
YL6-24	40.3 ± 5.1c	7.5 ± 0.0b	5017.5 ± 137.4d	7.0 ± 0.8c	5.5 ± 0.0d	2.3 ± 0.1c	2.9 ± 0.1c	10.1 ± 0.4c	4.2 ± 0.0c
YL6-GFP-24	29.1 ± 3.1c	7.6 ± 0.1b	2866.9 ± 201.1e	6.0 ± 0.5c	5.6 ± 0.2d	2.6 ± 0.2c	2.5 ± 0.2d	9.4 ± 0.3c	4.3 ± 0.0b
YL6-48	61.8 ± 4.9b	8.7 ± 0.4a	9861.8 ± 231.1a	25.6 ± 0.8b	21.6 ± 0.3a	3.0 ± 0.2b	3.9 ± 0.2a	13.6 ± 0.5a	4.0 ± 0.0d
YL6-GFP-48	54.6 ± 6.2b	8.7 ± 0.2a	9197.3 ± 212.6b	24.8 ± 1.2b	22.4 ± 0.7a	3.2 ± 0.1b	3.8 ± 0.1ab	12.9 ± 0.4a	4.0 ± 0.0d
YL6-72	139.0 ± 28.9a	7.8 ± 0.3ab	8210.3 ± 300.1c	37.1 ± 3.1a	15.9 ± 1.2c	4.0 ± 0.2a	3.8 ± 0.2ab	12.1 ± 0.3ab	5.2 ± 0.0a
YL6-GFP-72	112.9 ± 33.1a	8.1 ± 0.6ab	8161.1 ± 281.4bc	36.4 ± 2.6a	17.0 ± 1.5b	4.2 ± 0.2a	3.6 ± 0.2b	11.9 ± 0.7b	5.2 ± 0.0a

*Different lowercase letters indicate significant difference (*P* < 0.05).*

Among these acids, the contents of citric, acetic, and ascorbic acids were significantly correlated with the soluble P content (*R*^2^ = 0.850, *R*^2^ = 0.982, and *R*^2^ = 0.966, respectively, *P* < 0.05); the content of tartaric acid was significantly correlated with the activity of alkaline phosphatase (*R*^2^ = 0.943; *P* < 0.05) and the activity of alkaline phosphatase was positively correlated with the pH (*R*^2^ = 0.733, *P* < 0.05) and the content of soluble P (*R*^2^ = 0.839, *P* < 0.05).

### Effect of YL6 on Chinese Cabbage Plant Growth under Field Conditions

The effect of the application of YL6 in the field on growth parameters and nutrient uptake of Chinese cabbage plants is shown in **Table 2**. As the table shows, compared to the non-inoculated CK0, the plant height, root length, root volume, and leaf number were significantly increased (*P* < 0.05). Soil inoculated with YL6 promoted the root growth of Chinese cabbage plants. The root volume attained (1.7 mL) was significantly higher than that obtained with CK0 (0.5 mL) (Supplementary Table S1). **Table 2** shows that the fresh and dry weights of YL6 treated Chinese cabbage plants reached 24.3 and 2.3 g/plant, respectively, which were significantly higher than those in the CK1, CK0, and CK2 treatments. And we can clearly observe these difference in **Supplementary Figure S3**.

**Table 2:** Effects of YL6-GFP on the biomass of Chinese cabbage under field conditions.

Treatments	Fresh weight (g/plant)		Dry weight (g/plant)	
	Shoot	Root	Shoot	Root
CK0	6.1 ± 0.3d	0.6 ± 0.1d	0.7 ± 0.1d	0.1 ± 0.0c
CK1	15.6 ± 0.3c	1.3 ± 0.1c	1.5 ± 0.1c	0.2 ± 0.0b
CK2	18.1 ± 0.7b	1.6 ± 0.1b	1.7 ± 0.1b	0.2 ± 0.0b
YL6	24.3 ± 0.4a	2.4 ± 0.1a	2.3 ± 0.1a	0.4 ± 0.0a

*Different lowercase letters indicate significant difference (*P* < 0.05).*

### Influence of the Soil Physical and Chemical Properties and PSB Population in the Chinese Cabbage Rhizosphere

The effect of YL6 on the pH, soluble P, available potassium, nitrogen (nitrate and ammonium), organic matter, and the number of YL6 bacteria observed in the rhizosphere soil is presented in **Table 3**. PSB secrete organic acids to reduce the soil pH and promote the release of available P from calcareous soil, which is the primary P-solubilization mechanism (Kinraide et al., 2005; Wagh et al., 2014; Chen et al., 2015). After the application of YL6, the soil pH (8.3) was the lowest (**Table 3**), whereas the PSB count (6.0 lg CFU/g dry weight) was the highest, with 1.7% more PSB than in the control group (*P* < 0.05) (Supplementary Table S2). The content of soluble P in the soil (5.50 mg/kg) was higher than that in the CK0, CK1, and CK2 treatments. Therefore, the PSB effectively improved the content of available P in the soil, thus promoting the growth of the Chinese cabbage plants. In addition, after applying YL6, the soluble P, soluble potassium, nitrogen (nitrate and ammonium), and organic matter content were significantly higher than those in the CK0, CK1, and CK2 treatments. These results showed that PSB could improve the content of soluble P and improve soil fertility and Ilany and Ali’s researches also supported above results (Ilany et al., 2010; Ali et al., 2012).

**Table 3:** Influence of YL6 on soils nutrients for Chinese cabbage under field conditions.

Treatments	Soluble P mg/kg	Soluble potassium mg/kg	Nitrate nitrogen mg/kg	Ammonium nitrogen mg/kg	Soil organic matter g/kg	pH
CK0	4.3 ± 0.3c	130.7 ± 2.9d	4.8 ± 0.4d	26.7 ± 1.4c	25.9 ± 1.8c	8.3 ± 0.0b
CK1	4.7 ± 0.3b	151.3 ± 3.6b	6.6 ± 0.6b	29.0 ± 1.2ab	28.2 ± 1.5b	8.3 ± 0.0b
CK2	4.4 ± 0.3bc	142.9 ± 10.8c	5.7 ± 0.3c	28.2 ± 1.3a	26.5 ± 1.2bc	8.4 ± 0.1b
YL6	5.5 ± 0.4a	164.9 ± 4.1a	8.6 ± 0.6a	30.4 ± 1.6bc	30.3 ± 2.4a	8.3 ± 0.0a

*Different lowercase letters indicate significant difference (*P* < 0.05).*

### GFP-Expressing YL6

A GFP-expressing YL6 strain was constructed for histological studies. The *GFP* gene was introduced into the YL6 strain by triparental mating. The *E. coli* strain DH5α, carrying the target plasmid (pMP2444), and *E. coli* HB101, carrying the shuttle plasmid (pK2013), were used to introduce the *GFP* gene into the recipient YL6 strain. Then, we extracted plasmids inserted GFP and the recombination strain for PCR detection and observed a clear target band at 968 bp (data not show). So they are completely homologous to the known *GFP* gene. The new recombination strain was named YL6-GFP.

### Detection of GFP Expression in the YL6-GFP Strain

**FIGURE 1:**
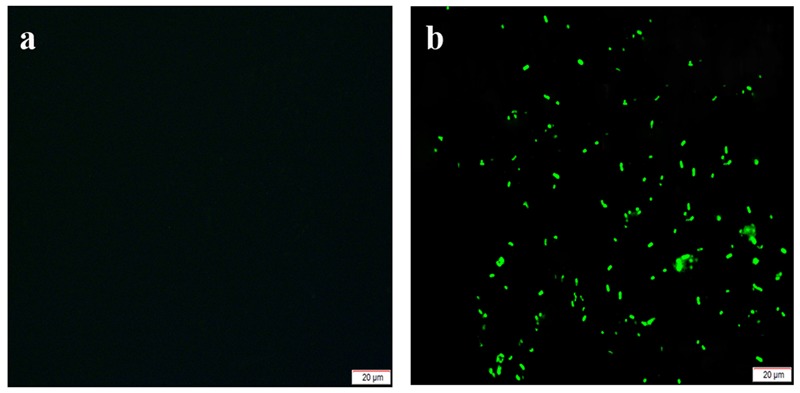
Fluorescence microscopy detection of the green fluorescent protein (GFP)-tagged strain YL6-GFP [**a**: original strain (Yangling 6, YL6); **b**: marked strain (YL6-GFP)].

Positive clones that were evenly coated on glass slides and observed by fluorescence microscopy were found to be short rods that emitted green fluorescence. Thus, the *GFP* gene was shown to have been successfully introduced and expressed in the YL6 strains (**Figure 1**).

### Comparison of the Biological Characteristics of the YL6 and YL6-GFP

#### Growth Curve

Both the YL6-GFP and parental YL6 strains remained in the lag phase during the first 3 h of growth (**Supplementary Figure S4**) and then spent 5 h in the logarithmic phase. After 8 h, the two strains reached the stationary phase, but YL6 grew slightly faster than the YL6-GFP strain during the logarithmic and stationary phases. This illustrated that there were some differences between two strains on cell growth resulting from the introduction of the plasmid, possibly due to little more energy consumption in the YL6-GFP strain. However, their growth curves had the same trend; therefore, the exogenous plasmid did not have a detrimental effect on cell growth.

#### Analysis of P-Solubilization Activity on an Inorganic P Liquid Medium

YL6-GFP was inoculated into inorganic P liquid medium. The content of soluble P in this type of media rose initially and then decreased. After 48 h, the content of soluble P reached 12.6 μg/mL (**Table 1**). During the same period of growth, the content of soluble P in the YL6-GFP media was consistently slightly lower than that of YL6, which indicated that the exogenous plasmid had a small effect on the activity compared to the parental strain.

The activity of alkaline phosphatase was also positively correlated with the pH of the GFP-expressing strain (*R*^2^ = 0.758, *P* < 0.05), and acid phosphatase and soluble P were positively correlated (*R*^2^ = 0.869, *P* < 0.05); the secretion of organic acids and the activity of acids or alkaline phosphatase of the GFP-expressing strain were not significantly different compared to the starting strain.

#### Colonization Dynamics of YL6-GFP in Natural Soil

To explore the colonization dynamics of YL6-GFP in natural soil, we set up different treatments to observe the number of YL6-GFP bacteria at different periods (**Supplementary Figure S5**). Because PSB must adapt to the external environment, the number of bacteria decreased with the increase in inoculation days. As **Supplementary Figure S5** shows, the bacterial density was highest at 0 days, which was the first day of inoculation. The number of YL6-GFP colonies decreased to 4.9–5.8 lg CFU/g dry weight on the 21st day and remained at this order of magnitude. Therefore, YL6-GFP could stably colonize in the natural soil and display similar growth dynamics to YL6.

#### Pot Experiment

**FIGURE 2:**
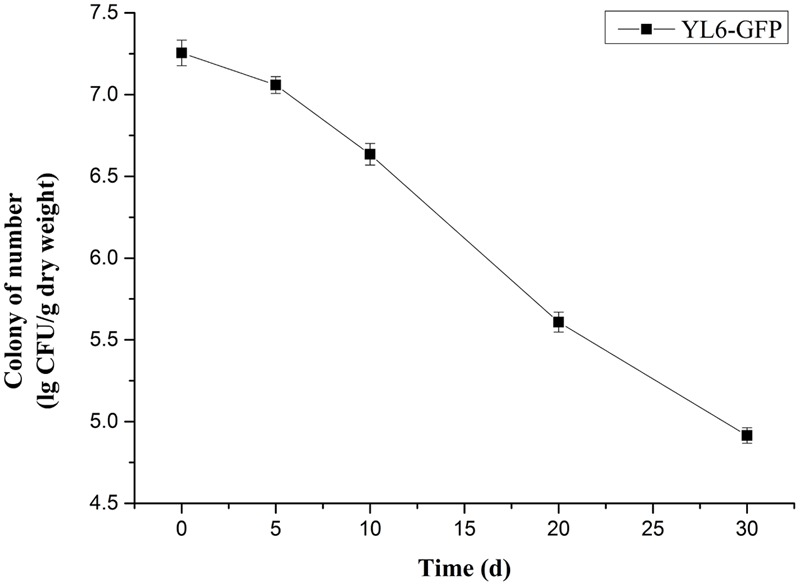
Colonization byYL6-GFP in the rhizosphere soil of Chinese cabbage.

The soil soluble P content with the YL6-GFP treatment increased to 14.2 mg/kg and displayed no obvious differences compared to YL6 (*P* < 0.05); moreover, it was significantly higher than that of CK1 (13.5 mg/kg) and CK0 (12.7 mg/kg) (*P* < 0.05) (Supplementary Table S3). The ample P content in the soil promoted root growth, resulting in root expansion into more soil space for assimilating nutrient uptake. The roots of Chinese cabbage treated with YL6-GFP and YL6 were more developed and had root lengths of 12.5 and 12.7 cm, respectively, which were significantly longer than roots with the CK0 and CK1 treatments. A developed root system is beneficial to the development of aboveground parts. The plant height, leaf number, root length, and root volume of the YL6-GFP treated plants increased 16.5, 34.9, 27.6, and 260.0%, respectively, compared to CK0 (Supplementary Table S3). After the application of YL6-GFP, a single plant weight was 18.5 g higher compared to CK0, whereas the yield of individual plants had increased by 147.4% (*P* < 0.05) (Supplementary Table S4). Meanwhile, in the samples treated with YL6, the aboveground fresh and dry weight and the underground fresh and dry weight were similar to those in the YL6-GFP treatment, which illustrated that the introduction of the plasmid did not affect the actual application of PSB. In the pot experiment, the number of YL6-GFP in the rhizosphere soil of Chinese cabbage decreased over time. The early stage of inoculation displayed the highest number of YL6-GFP (7.3 lg CFU/g at 0 days), but in the rhizosphere soil of Chinese cabbage, the concentration of YL6-GFP was still approximately 4.9 lg CFU/g at 30 days (**Figure 2**).

#### Observations of Chinese Cabbage Radicle Treated by YL6-GFP

**FIGURE 3:**
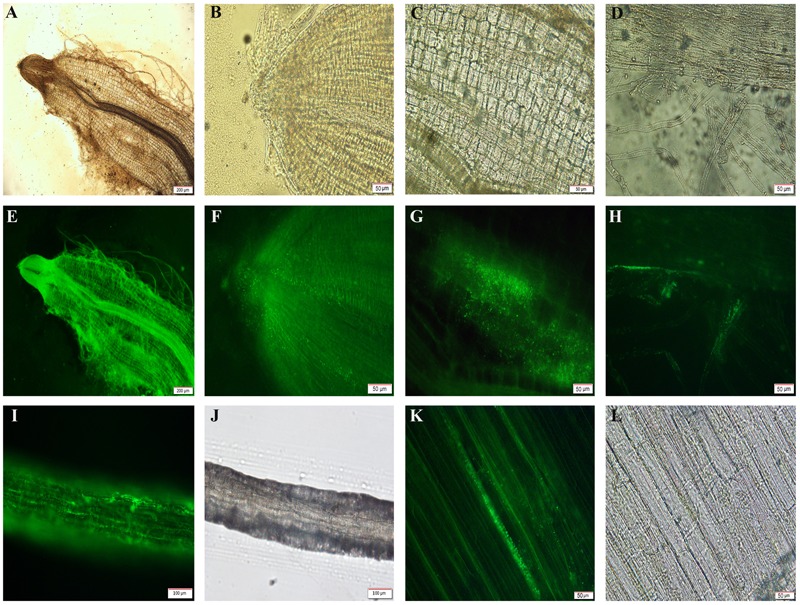
Fluorescence micrograph of the different tissues of Chinese cabbage colonized by YL6-GFP (**A,E,B,F**, root tip; **C,G,D,H**, root hairs; **I,J**, epicotyl; **K,L**, hypocotyl epidermis).

YL6-GFP was frequently observed to colonize the surface of the cabbage root cap and root epidermal cells on the first or third days (**Figures 3A,E**). During the process of root tip growth, the extension of pileorhiza is constant, and root elongation is continuous. There were many marked strains gathered in the root cap, because the new cytoderm was so thin that YL6-GFP easily entered the root. A local enlarged photo of the root cap showed that YL6-GFP had moved into its tissue (**Figures 3B,F**). The two pictures indicated that the labeled bacteria primarily entered the root system via the root cap, which might be the hotspot for YL6-GFP colonization. Many YL6-GFP were found at the intercellular space and intracellular region of the apical meristem zone (**Figures 3C,G**). Simultaneously, a motion trail of GFP-expressing bacteria were clearly observed in root hair, where cells are long and the cell walls are thin (**Figure 3G**). The lifecycle of root hair is short. Root hair could enhance the absorption area and secrete acid to help roots take in nutrients, which can transport water and nutritive material into other above ground parts of the plant. There were large number of YL6-GFP in the epicotyl (**Figures 3D,H**). This phenomenon suggested that GFP-expressing strains might be carried by water (**Figures 3I,J**) to other plant tissues. Then we made further observations of epidermal cells at higher magnification (**Figures 3K,L**). A significant amount of YL6-GFP were found in many epidermal cells of the epicotyl. We could infer from the above results that YL6-GFP entered the Chinese cabbage root tissue through the gap of root epidermal cells in the root hair and root cap and was then transported by water in the longitudinal direction to hypocotyl vascular tissue through the vascular bundle in the root.

#### Mobility of YL6-GFP in Chinese Cabbage

As **Table 4** shows, YL6-GFP could invade the root quickly during the first week when we began the sterile seedling test. Increasing numbers of GFP-expressing cells were found in root tissues, and the number of GFP-expressing bacteria increased to 4.5 lg CFU/g dry weight and 4.3 lg CFU/g dry weight in the rhizosphere soil during the third week. Then, the strains moved to leaves in the third week.

**Table 4:** Colonization dynamics of YL6-GFP in leaves, roots and rhizosphere soil of Chinese cabbage.

	Colonization number of YL6-GFP (lg CFU/g dry weight)		
	1 week	2 weeks	3 weeks
Rhizosphere soil	4.2	4.3	4.3
Roots	3.3	4.2	4.5
Leaves	0	0	3.9

## Discussion

P is an important factor limiting agricultural production (Goldstein, 1951; Asea et al., 1988). Large amounts of P fertilizer added to soil not only increase the cost of agriculture but also cause environmental problems (Shahid et al., 2012; Hanif et al., 2015; Majeed et al., 2015) and are not conducive to the sustainable development of agriculture. PSB have a great ability to transform insoluble P in the soil into an available form and have great application prospects for eco-agriculture. The secretion of organic acids and chelation are major P-solubilization mechanisms of PSB. The presence of many PSB in the soil is an important index of effective promotion of crop growth and sustainable agricultural development (Fernández et al., 2012).

We isolated YL6 from the rhizosphere of Chinese cabbage as a bacterium with a strong potential to solubilize phosphate. The bacterium was identified as *B. cereus* by 16S rRNA sequence analysis, which is a common method used to identify bacterial species (Clarridge, 2004; Coelho et al., 2011). Based on the analysis of the P-solubilization of YL6, the P-solubilization was the highest at 48 h. Recent studies also reported that the content of soluble P does not change over time (Zhao et al., 2014; Li et al., 2015; Osorio and Habte, 2015). This might be because PSB can secrete organic acids to degrade TCP to promote the content of soluble P in culture medium; thus, the content of soluble P does not decrease over time. With the increase in culture time and nutrient consumption, the insoluble P in the culture is converted to a form that plants can absorb (Chatli et al., 2008). Therefore, the P-solubilization activity declines after peaking. The number of PSB and the culture pH were found to be negatively proportional with different culture times (*R*^2^ = -0.737, *P* < 0.05), and the content of soluble P was positively correlated with acid phosphatase activity (*R*^2^ = 0.839, *P* < 0.05), which was also demonstrated in research by Chen and Shahid (Chen et al., 2006; Shahid et al., 2012).

In the field experiment, because the principal mechanism for P-solubilization in soil is lowering the soil pH via the PSB production of organic acids (Chatli et al., 2008; Badawi, 2010), we found that soluble P in the soil increased significantly after inoculation with YL6, and the soil pH decreased markedly. The content of nitrogen (nitrate and ammonium) and organic matter also increased, indicating that PSB could improve soil nutrients. In addition, P promotes the development of roots; thus, the growth of Chinese cabbage roots was significantly improved after the application of YL6 (Chen et al., 2017). The root is the main organ for absorbing minerals and water and participates in material transport (Shane et al., 2003; Heppell et al., 2015). Therefore, it is easy to understand that the fresh and dry plant weight and plant height were significantly increased with the improved root system. These findings corroborate earlier reports on plants such as the sunflower (*Helianthus annuus*), cucumber (*Cucumis sativus* L.), and potato (*Solanum tuberosum* L.) (Shahid et al., 2014; Hanif et al., 2015).

Green fluorescent protein and other fluorescence markers are valuable tools, allowing colonization by recombinant microorganisms to be visualized via fluorescence microscopy (Luby-Phelps et al., 2003). To obtain a stable genetic transformation, the target gene was introduced into YL6 by a triparental mating (Liu et al., 2012). The GFP-expressing YL6 strain (YL6-GFP) was similar to the original strain (YL6), both in its growth and ability to dissolve P. The ability of PSB survival in the soil is the key to determining its role in the natural environment and is an important basis on which to evaluate PSB colonization ability in the rhizosphere (Shen et al., 2013; Hanif et al., 2015). To detect and quantify PSB colonization in natural (non-sterile) soil, we used a dilution series method and antibiotic resistance to determine the growth and colonization of the bacteria (Brown and Waatti, 1980). In the inoculation experiments in natural soil, the YL6-GFP population remained stable for up to 21 days (5.0–5.8 lg CFU/g of dry soil) after the initial decrease in population density. In the experimental pots, the rhizosphere soil of Chinese cabbage still contained approximately 4.9 lg CFU/g after 30 days. It is possible that YL6 quickly established a stable interaction with the host plant roots. Indeed, the reason why the number of PSB was higher than that of other microorganisms in soils is that the presence of the host plant may have greatly affected the survival of PSB that were attracted to the rhizosphere of the growing seedlings (Bashan et al., 1995; Bonaldi et al., 2015). In the pot experiments, YL6-GFP did not display a negative influence on the growth of Chinese cabbage plants. Thus, YL6-GFP and YL6 did not affect this practical application result.

To further explore the mechanism of growth-promoting bacteria, we used fluorescence microscopy to study the distribution of GFP-expressing strains in the roots of Chinese cabbage and the change in the presence of YL6-GFP (Coombs and Franco, 2003; Olivain et al., 2006; Fernández et al., 2012). The fluorescence labeling technique is widely used to observe the colonization of microorganisms in roots or root tips (Schippers et al., 1995; Simons et al., 1996; Anand and Chanway, 2013; Zhao et al., 2013; Chen et al., 2016). There is much research on the colonization of biocontrol bacteria of plants, but little on PSB. Lelis et al. (2014) noted that most *Clavibacter michiganensis* sub sp. IPO3356 entered tomato (*Lycopersicon esculentum* Mill.) plant tissues through wounds or during the formation period. Surprisingly, 28–30°C is the most suitable condition for microbial activity. The PSB (Ps-5) was also found in the root tissue of the sunflower by Shahid, indicating that Ps-5 might be an endophytic bacterium that promotes crop growth and can enter plant tissue. However, there has not been a long-term study of its ability to survive in the rhizosphere (Shahid et al., 2014). For *Bacillus subtilis* P2b-2R, when a GFP-expressing strain (P2b-2Rgfp) was applied to canola (*Brassica campestris* L.) and tomato, P2b-2Rgfp was also observed in the roots and leaves of seedlings 20, 30, and 40 days after treatment. GFP-labeled P2b-2R did not affect its biological activity (Padda et al., 2015). A PSB strain that was isolated from soil and engineered to express GFP was also studied by Fernández et al. (2012). The number of colonizing P bacteria ranged from 1.7 × 10^7^ to 7.6 × 10^5^ CFU/g over a 0–15 days period. These authors detected fluorescence labeling in the rhizosphere bacteria but did not conduct further monitoring (Fernández et al., 2012). In our study, YL6-GFP could survive in the natural soil, rhizosphere soil, and roots. YL6-GFP could rapidly colonize the Chinese cabbage root and hypocotyl cells. These phenomena indicated that YL6 might be endophytic, able to enter the plant tissue, and establish interactions with the host to promote plant growth. To further validate our ideas, we cultivated Chinese cabbage under sterile conditions and then found that the biomass significantly increased at 1-, 2-, and 3-weeks, with many YL6-GFP bacteria present in the root tissue.

The colonization ability of microorganisms on roots has a significant influence on establishing an interaction relationship with the host (Chen et al., 2016). PSB can turn insoluble P into soluble P and promote plant growth (Rodríguez et al., 2006; Zhao et al., 2014). The promotion of plant growth by PSB has been confirmed by data gathered in the field and in indoor pot tests. By studying the colonization capability of YL6 on Chinese cabbage using a GFP-expressing YL6 strain, we found that GFP-expressing bacteria were present in the tissue of the Chinese cabbage plants. This research helps us to understand the advantage that PSB provide in plant growth at the cellular level. Research on the colonization of YL6 in Chinese cabbage tissues provides a theoretical foundation for understanding the P-solubilization mechanism of PSB and offers a basis for the application and development of eco-friendly, high-yield agriculture.

## Author Contributions

ZW isolated strain YL6, marked strains by GFP, cultivated Chinese cabbages in pot with application of YL6 and YL6-GFP, observed colonization of Chinese cabbages seedling in sterile condition, and drafted the manuscript. GX cultivated Chinese cabbages in field condition and helped ZW finish original manuscript. XY helped ZW use fluorescence microscope take useful photos. CC participated in the discussions of each section of experiments, designed the outline of this and partly supported this research. YL supported ZW to build YL6-GFP in her lab. PM improved manuscript.

## Conflict of Interest Statement

The authors declare that the research was conducted in the absence of any commercial or financial relationships that could be construed as a potential conflict of interest.

## Supplementary Material

FIGURE S1 Gram stain of YL6.

FIGURE S2 Formation of a phosphate-solubilizing zone around the YL6 (left) and YL6-GFP (right) strains.

FIGURE S3 Morphology of Chinese cabbage after the application of YL6 under field conditions.

FIGURE S4 Growth curves of YL6 and YL6-GFP.

FIGURE S5 Colonization of the YL6-GFP strain in soil (**A**: 3% inoculum size; **B**: 6% inoculum size; **C**: 9% inoculum size).
