# Supplementary figures and images for: Isolation and Characterization of a Phosphorus-Solubilizing Bacterium from Rhizosphere Soils and Its Colonization of Chinese Cabbage (Brassica campestris ssp. chinensis)
Source: Front Microbiol. 2017 Jul 26;8:1270. doi: 10.3389/fmicb.2017.01270 (PMC5526974; doi:10.3389/fmicb.2017.01270)

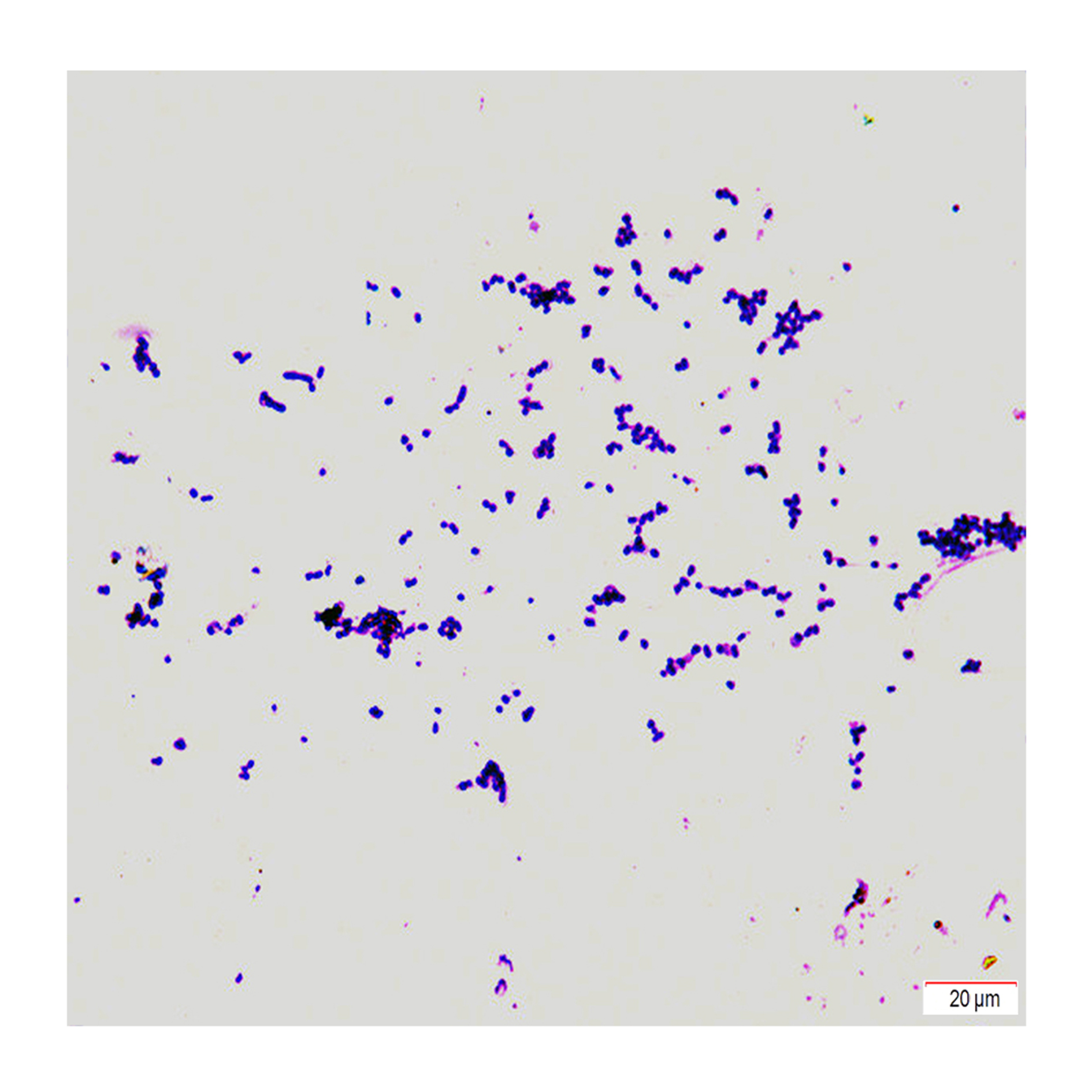

Supplement: FIGURE S1 — Gram stain of YL6. [file Image_1.JPEG]

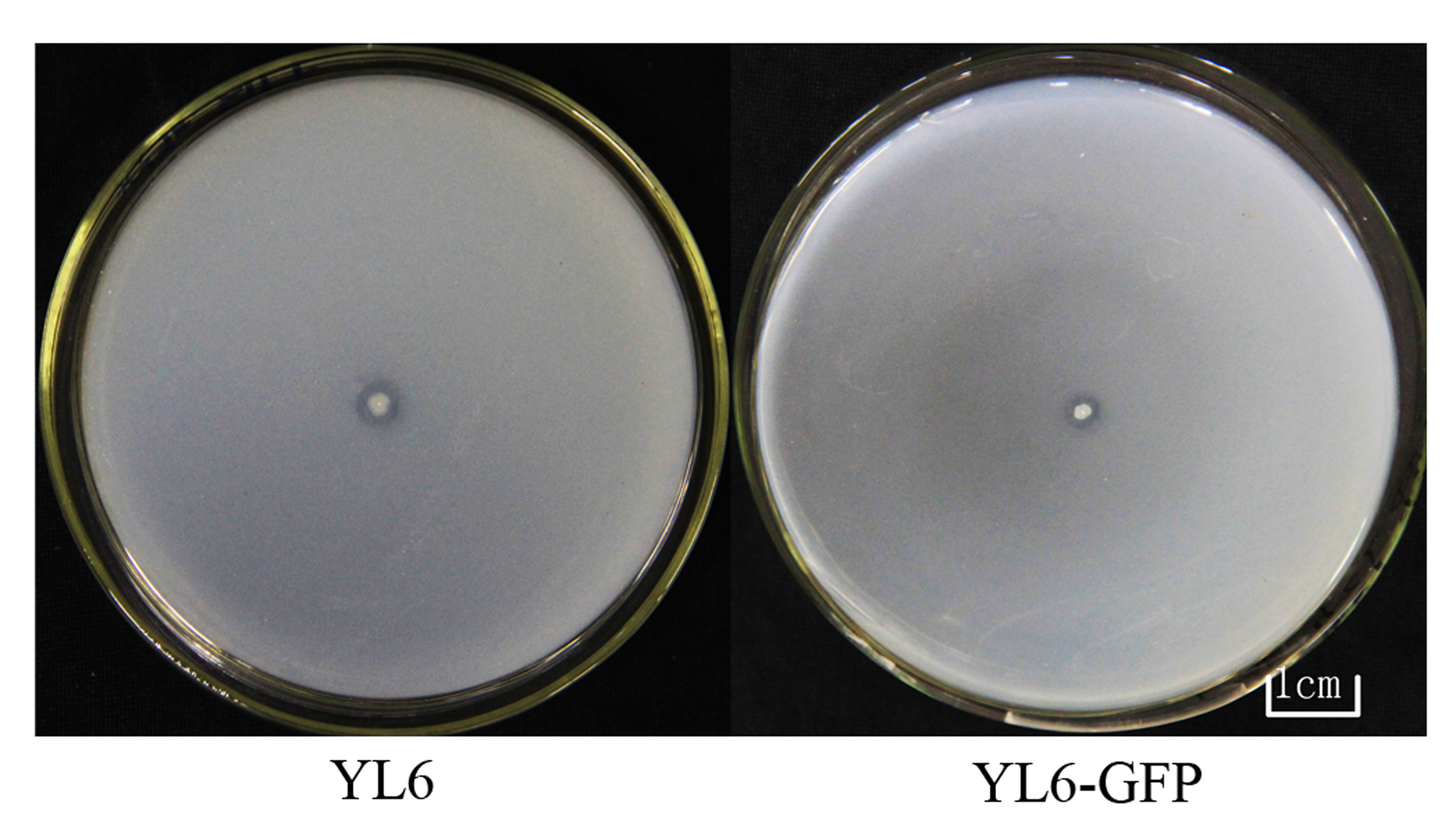

Supplement: FIGURE S2 — Formation of a phosphate-solubilizing zone around the YL6 (left) and YL6-GFP (right) strains. [file Image_2.JPEG]

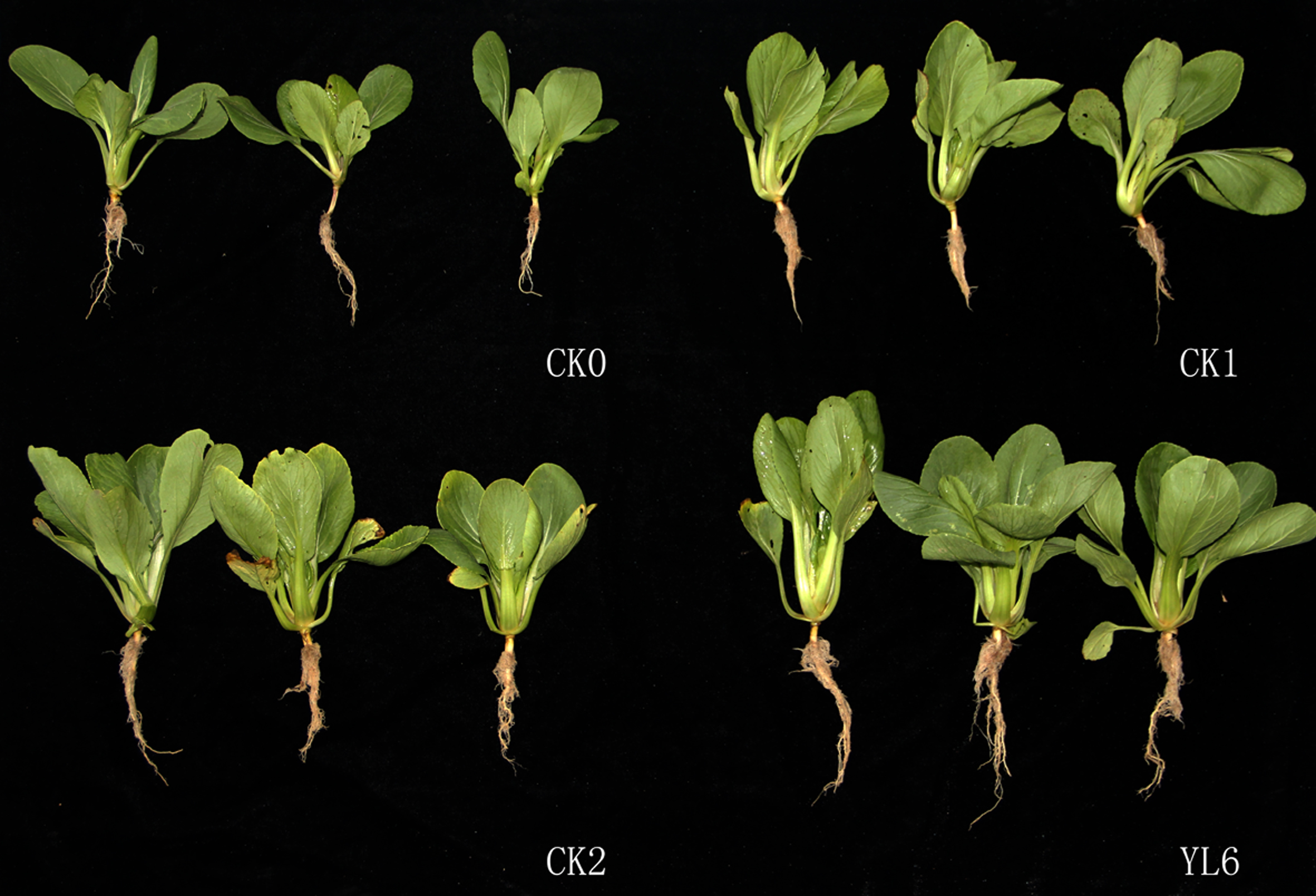

Supplement: FIGURE S3 — Morphology of Chinese cabbage after the application of YL6 under field conditions. [file Image_3.JPEG]

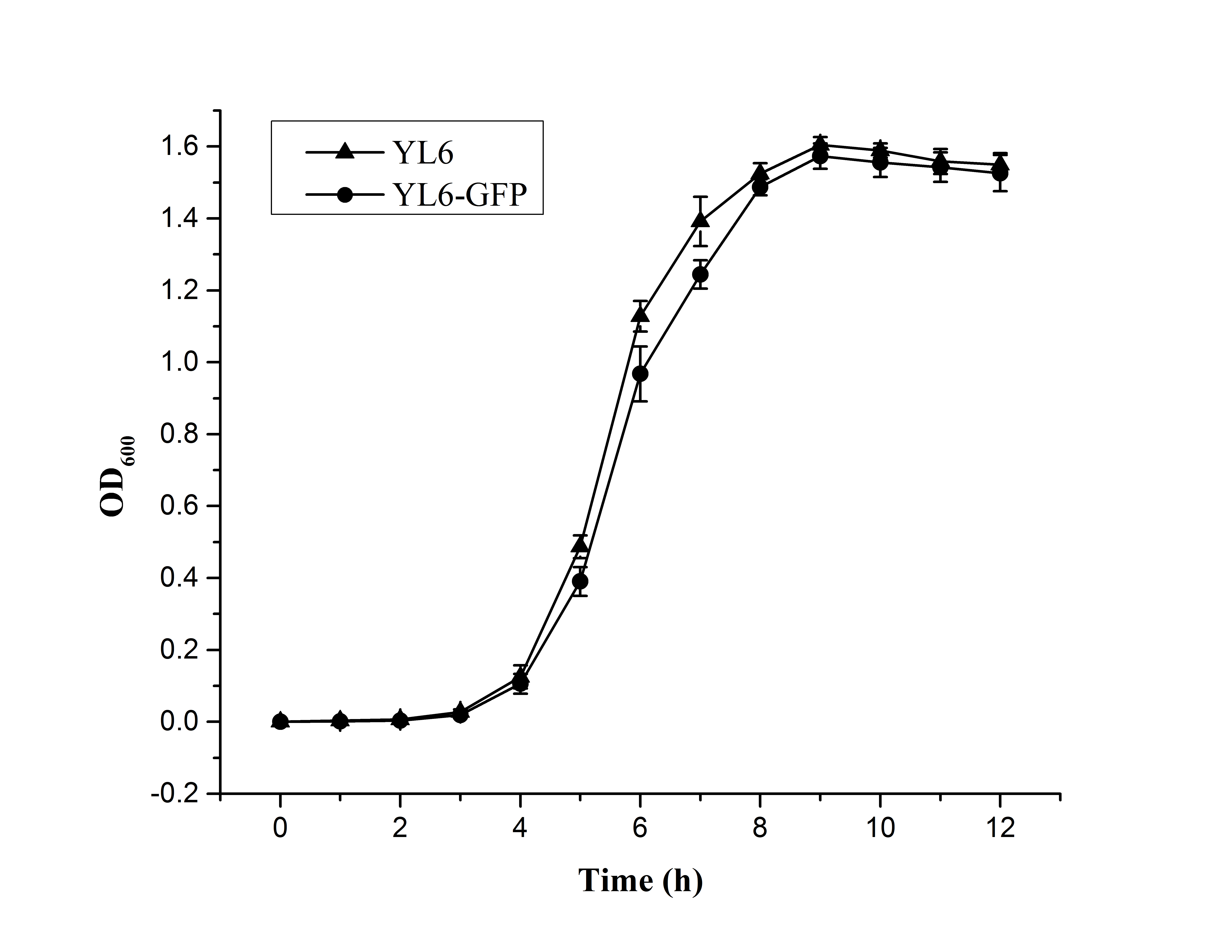

Supplement: FIGURE S4 — Growth curves of YL6 and YL6-GFP. [file Image_4.jpg]

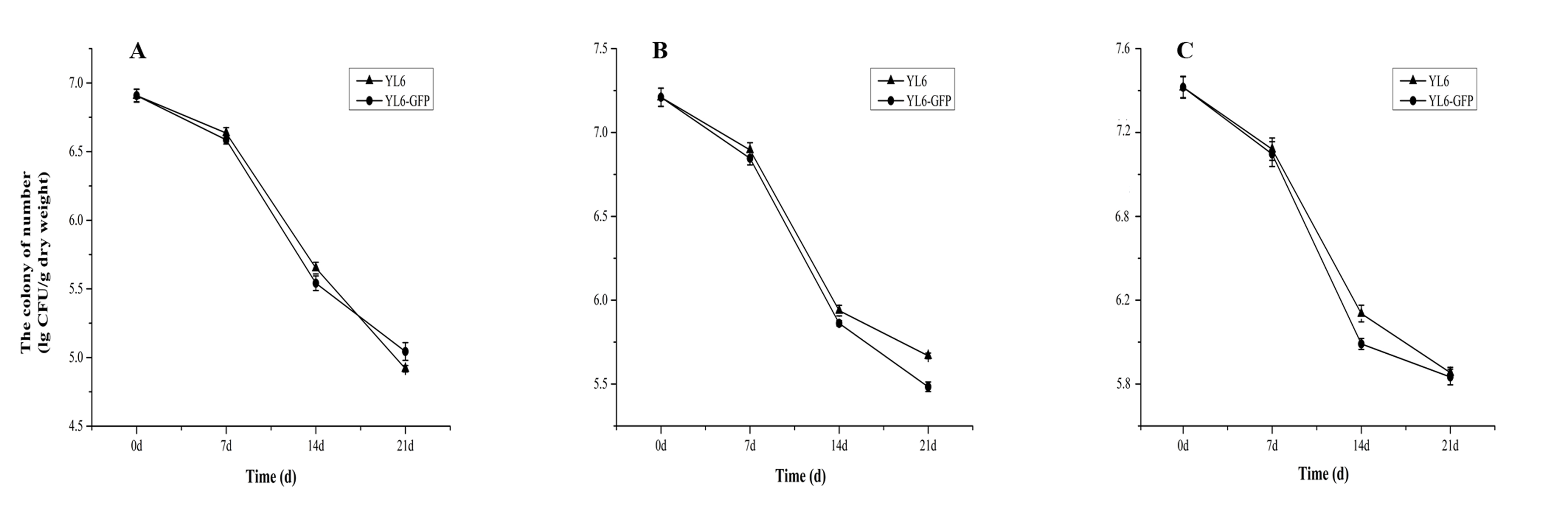

Supplement: FIGURE S5 — Colonization of the YL6-GFP strain in soil (A: 3% inoculum size; B: 6% inoculum size; C: 9% inoculum size). [file Image_5.JPEG]
