# Supplementary material for: Isolation and Characterization of a Phosphorus-Solubilizing Bacterium from Rhizosphere Soils and Its Colonization of Chinese Cabbage (Brassica campestris ssp. chinensis)
Source: Front Microbiol. 2017 Jul 26;8:1270. doi: 10.3389/fmicb.2017.01270 (PMC5526974; doi:10.3389/fmicb.2017.01270)
Supplement: Supplementary file 6 [file Table_1.DOCX]

## 1.2 Supplementary Tables

**SUPPLEMENTARY TABLE1.** Effects of YL6-GFP on agronomic characters of Chinese cabbage under field conditions

| Treatments | Plant height | Root length | Leaf number | root volum |
| --- | --- | --- | --- | --- |
|  | cm | cm |  | ml |
| CK0 | 12.8±0.6c | 10.3±0.3c | 6.8±0.5c | 0.5±0.1d |
| CK1 | 13.2±0. 5bc | 9.4±1.0c | 8.0±0.6b | 1.3±0.4c |
| CK2 | 14.2±0.6b | 12.9±1.0b | 8.3±0.6b | 1.5±0.1b |
| YL6 | 16.3±0.8a | 15.3±1.0a | 11.8±1.0a | 1.7±0.1a |

Different lowercase letters indicate significant difference (*P*<0.05), the same below
