# Supplementary material for: Isolation and Characterization of a Phosphorus-Solubilizing Bacterium from Rhizosphere Soils and Its Colonization of Chinese Cabbage (Brassica campestris ssp. chinensis)
Source: Front Microbiol. 2017 Jul 26;8:1270. doi: 10.3389/fmicb.2017.01270 (PMC5526974; doi:10.3389/fmicb.2017.01270)
Supplement: Supplementary file 7 [file Table_2.docx]

**Supplementary Table2** Effect of PSB on the total number of colonies and the number of phosphate-solubilizing bacteria of rhizosphere soil.

| Treatments | Total Bacteria  (1g CFU/g dry weigh) | Phosphate-solubilizing Bacteria  （lg CFU/g dry weigh） |
| --- | --- | --- |
| CK0 | 7.0 | 5.9 |
| CK1 | 7.2 | 5.9 |
| CK2 | 6.8 | 5.9 |
| YL6 | 7.2 | 6.0 |
