# Supplementary material for: Isolation and Characterization of a Phosphorus-Solubilizing Bacterium from Rhizosphere Soils and Its Colonization of Chinese Cabbage (Brassica campestris ssp. chinensis)
Source: Front Microbiol. 2017 Jul 26;8:1270. doi: 10.3389/fmicb.2017.01270 (PMC5526974; doi:10.3389/fmicb.2017.01270)
Supplement: Supplementary file 8 [file Table_3.docx]

**Supplementary Table 3** Effects of YL6-GFP on agronomic characters of Chinese cabbage and the content souble P of soil in pot experiment

| Treatments | Plant heights | Root length | Root volume | Blade number | Souble P |
| --- | --- | --- | --- | --- | --- |
|  | (cm) | (cm) | (mL) |  | (mg/kg) |
| CK0 | 12.1±0.1b | 9.8±0.3c | 0.5±0.0c | 6.3±0.6c | 12.8±0.2c |
| CK1 | 12.5±0.3b | 10.8±0.1b | 1.2±0.1b | 7.3±0.6ab | 13.5±0.3b |
| YL6 | 14.2±0.6a | 12.7±0.3a | 1.9±0.1a | 8.6±0.6a | 13.8±0.5a |
| YL6-GFP | 14.1±0.4a | 12.5±0.2a | 1.8±0.0a | 8.5±0.6a | 14.2±0.6a |

(four treatments in pot experiments: fermentation broth of YL6 (YL6), fermentation broth of YL6-GFP (YL6-GFP), pure pikovskaya’s medium(CK1) and H_2_O (CK0))
