# Supplementary material for: Isolation and Characterization of a Phosphorus-Solubilizing Bacterium from Rhizosphere Soils and Its Colonization of Chinese Cabbage (Brassica campestris ssp. chinensis)
Source: Front Microbiol. 2017 Jul 26;8:1270. doi: 10.3389/fmicb.2017.01270 (PMC5526974; doi:10.3389/fmicb.2017.01270)
Supplement: Supplementary file 9 [file Table_4.docx]

**Supplementary Table4** Effects of YL6-GFP on the biomass of Chinese Cabbage under pot culture

| Treatments | Fresh weight (g/plant) | | | Dry weight (g/plant) | | |
| --- | --- | --- | --- | --- | --- | --- |
|  | Shoot | Root | Plant | Shoot | Root | Plant |
| CK0 | 6.7±0.5c | 0.9±0.1c | 7.6±0.5c | 0.7±0.0c | 0.1±0.0c | 0.8±0.0c |
| CK1 | 12.0±0.7b | 1.3±0.2b | 13.3±0.6b | 1.4±0.1b | 0.1±0.0b | 1.5±0.1b |
| YL6 | 16.1±0.6a | 2.4±0.2a | 18.5±0.7a | 1.9±0.1a | 0.2±0.0a | 2.1±0.1a |
| YL6-GFP | 16.2±0.4a | 2.6±0.2a | 18.8±0.4a | 1.8±0.1a | 0.2±0.0a | 2.0±0.0a |
